# Rotigotine transdermal system as add-on to oral dopamine agonist in advanced Parkinson’s disease: an open-label study

**DOI:** 10.1186/s12883-015-0267-7

**Published:** 2015-02-28

**Authors:** Jong-Min Kim, Sun Ju Chung, Jae Woo Kim, Beom Seok Jeon, Pritibha Singh, Stephan Thierfelder, Junji Ikeda, Lars Bauer, on behalf of the Asia Pacific Rotigotine Add-on Study Group

**Affiliations:** Department of Neurology, Seoul National University Bundang Hospital, Seoul National University College of Medicine, Seongnam-si, Republic of Korea; Department of Neurology, Asan Medical Center, University of Ulsan College of Medicine, Seoul, Republic of Korea; Department of Neurology, Dong-A University Medical Center, Busan, Republic of Korea; Department of Neurology, Seoul National University Hospital, Seoul National University College of Medicine, Seoul, Republic of Korea; UCB Pharma, Alfred-Nobel-Str 10, 40789 Monheim am Rhein, Germany; Otsuka Pharmaceutical Company, Ltd., Tokyo, Japan

**Keywords:** Advanced Parkinson’s disease, Dual therapy, Rotigotine transdermal system, Oral dopamine receptor agonist, Safety

## Abstract

**Background:**

Achieving optimal symptom control with minimal side effects is a major goal in clinical practice. Dual-agent dopamine receptor agonist (DA) therapy in Parkinson’s disease (PD) may represent a promising approach to treatment, as the combination of different pharmacokinetic/pharmacological profiles may result in a lesser need for high dosages and, accordingly, may be well tolerated. The objective of the current study was to investigate safety and efficacy of rotigotine transdermal system as add-on to oral DA in patients with advanced PD inadequately controlled with levodopa and low-dose oral DA.

**Methods:**

PD0015 was an open-label, multinational study in patients with advanced-PD and sleep disturbance or early-morning motor impairment. Patients were titrated to optimal dose rotigotine (≤8 mg/24 h) over 1–4 weeks and maintained for 4–7 weeks (8-week treatment). Dosage of levodopa and oral DA (pramipexole ≤1.5 mg/day, ropinirole ≤6.0 mg/day) was stable. Primary variable was Clinical Global Impressions (CGI) item 4: side effects, assessing safety. Other variables included adverse events (AEs), Patient Global Impressions of Change (PGIC), Unified Parkinson’s Disease Rating Scale (UPDRS) II and III, Parkinson’s Disease Sleep Scale (PDSS-2), Pittsburgh Sleep Quality Index (PSQI), and “off” time.

**Results:**

Of 90 patients who received rotigotine, 79 (88%) completed the study; 5 (6%) withdrew due to AEs. Most (83/89; 93%) had a CGI-4 score <3 indicating that rotigotine add-on therapy did not interfere with functioning; 6 (7%) experienced drug-related AEs that interfered with functioning (score ≥3). AEs occurring in ≥5% were application site pruritus (13%), dizziness (10%), orthostatic hypotension (10%), nausea (8%), dyskinesia (8%), and nasopharyngitis (6%). Numerical improvements in motor function (UPDRS III), activities of daily living (UPDRS II), sleep disturbances (PDSS-2, PSQI), and reduction in “off” time were observed. The majority (71/88; 81%) improved on PGIC.

**Conclusions:**

Addition of rotigotine transdermal system to low-dose oral DA in patients with advanced-PD was feasible and may be associated with clinical benefit.

**Trial registration:**

ClinicalTrials.gov identifier NCT01723904. Trial registration date: November 6, 2012.

**Electronic supplementary material:**

The online version of this article (doi:10.1186/s12883-015-0267-7) contains supplementary material, which is available to authorized users.

## Background

Treatment with chronic levodopa for the symptoms of advanced Parkinson's disease (PD) is often associated with the development of motor fluctuations and dyskinesia, which gradually worsen as the disease progresses [1,2]. These limitations of levodopa therapy have been managed by the use of concomitant treatment with non-ergot derived dopamine receptor agonists (DAs). The DAs have some advantages over levodopa, including longer half-lives [3], which may reduce or delay the onset of motor complications. Achieving optimal symptom control with minimal side effects is a major goal in clinical practice. Dual-agent DA therapy in PD may represent a promising approach to treatment, as the combination of different pharmacokinetic/pharmacological profiles may result in a lesser need for high dosages and, accordingly, may be well tolerated [4-6].

Rotigotine is a non-ergot derived DA with activity across D1 through D5 receptors as well as select adrenergic and serotonergic sites [7]. Continuous transdermal delivery of rotigotine maintains stable plasma levels over 24 hours with a single daily application [8], thus avoiding plasma level peaks and troughs associated with more pulsatile oral drug delivery. Symptoms of some PD patients may not be adequately controlled over the entire 24-h range with existing oral DA treatment. Since rotigotine transdermal system maintains stable plasma concentration over 24 h, add-on rotigotine may supplement the effects of oral DAs. Activation of the D1 receptor is unique to rotigotine among the non–ergot-derived DAs; pramipexole and ropinirole have been shown to act at the D2 and D3 receptors, but exhibit little or no affinity at the D1 receptor [9,10]. A synergistic interaction may exist between D1 and D2 receptors; a D1 receptor agonist has been shown to act synergistically with a D2 receptor agonist to prolong the motor stimulation induced by each agonist alone in the MPTP-lesioned monkey model of PD [11]. Thus, as a result of their different pharmacokinetic/pharmacological properties, there may be benefits of dual treatment with transdermally delivered rotigotine and oral ropinirole or pramipexole. Significant treatment effects of rotigotine transdermal system have been observed in double-blind, placebo-controlled studies as add-on therapy to levodopa in advanced-stage PD [12,13] (improvements in motor fluctuations; i.e., “on” and “off” time), and also in patients with PD and unsatisfactory control of early-morning motor function (improvements in early-morning motor function and sleep disturbance [a non-motor symptom of PD]) [14]. In addition, improvements in motor function and motor fluctuations have been demonstrated with 3-times-daily oral immediate-release (IR) ropinirole or pramipexole, or their once-daily oral extended-release (ER) formulations in combination with levodopa in patients with advanced PD [15,16].

The objective of this study was to investigate the safety and efficacy of rotigotine transdermal system as add-on to therapy with low-dose pramipexole or ropinirole, in patients with advanced PD. Enrolled patients were insufficiently controlled with levodopa and low-dose oral DA, identified as experiencing motor complications and sleep disturbance or early-morning motor impairment.

## Methods

### Patients

Patients enrolled in the PD0015 study included men and women, aged 30–80 years, with idiopathic PD of longer than 3 years’ duration, and Hoehn and Yahr stage II-IV. PD was defined by the presence of bradykinesia and at least one of the following: resting tremor, rigidity, or impairment of postural reflexes. In addition, all patients included had to be taking levodopa (immediate or controlled release, and at a stable dose) in combination with benserazide or carbidopa, and a stable low dose of IR or ER pramipexole (≤1.5 mg/day) or ropinirole (≤6.0 mg/day) for at least 28 days before baseline. Patients had to experience motor fluctuations (“wearing off”, “on-off” phenomena, delayed “on” or “non-on”) or dyskinesia, sleep disturbance or early-morning motor impairment, as determined by the investigator, and nocturia for at least 3 nights within 7 days before baseline. At the screening visit, patients were instructed to differentiate between “off” and “on” states and symptoms of “troublesome dyskinesia” for diary recordings. Patients completed 7 days of diary recordings before beginning study treatment; four of the diaries had to be determined “valid” by the investigator for the patient to be eligible. Permitted PD medications included anticholinergics, monoamine oxidase B (MAO-B) inhibitors, N-methyl-D-aspartate (NMDA) antagonists (e.g., amantadine), and entacapone; permitted CNS active drugs included sedatives, antidepressants, anxiolytics, hypnotics. All permitted drugs were required to be at stable doses for at least 28 days prior to baseline, and were to remain stable for the duration of the study.

Patients with clinically significant hepatic or renal impairment were excluded. Prohibited medications included DAs other than pramipexole or ropinirole, MAO-A inhibitors, dopamine-releasing substances, dopamine-modulating substances, tolcapone, budipine, dopamine receptor antagonists (antiemetics [other than domperidone, e.g., metoclopramide], and neuroleptics). The study was conducted in accordance with Good Clinical Practice and the Declaration of Helsinki. The study protocol and amendments were approved by the Institutional Review Board of all 21 centers (Additional file 1: Table S1). All patients provided written, informed consent before study participation.

### Study design and procedures

PD0015 (ClinicalTrials.gov:NCT01723904) was a Phase IIIb, open-label, single-arm study in patients with advanced PD (between October 2012 and April 2013). Twenty one centers enrolled patients, who applied at least one rotigotine patch, in South Korea, Malaysia, Taiwan, Australia, and Singapore.

Baseline data were recorded following a screening period of up to 4 weeks. Rotigotine was administered as once-daily patches of three different sizes; patches provided nominal doses of rotigotine 2 mg/24 h (10 cm^2^), 4 mg/24 h (20 cm^2^), or 6 mg/24 h (30 cm^2^). The study consisted of a 1-4-week titration and 4-7-week maintenance period (total of 8-week treatment); patients were titrated in weekly increments of 2 mg/24 h rotigotine to their optimal (or maximal allowed) dose (up to 8 mg/24 h) (Additional file 2: Figure S1). The optimal dose was defined as the dose at which both the investigator and patient felt that sleep disturbance or early-morning motor impairment was controlled. As this was the first study of rotigotine used concomitantly with a DA, the permitted upper doses were considered based on safety and the potential for overdose from concurrent use of DAs, in reference to the equivalent dosing regimens (Additional file 3: Table S2) [4,12,17]. The upper limit was determined as 1.5 mg/day for pramipexole (approved dose 4.5 mg/day), 6 mg/day for ropinirole (approved dose 24 mg/day), and 8 mg/24 h for rotigotine (EU-approved dose 16 mg/24 h). Using these upper dose limits, the combined total DA dose would not exceed the maximum approved dose of any of the DAs (see the equivalent dosing regimens in Additional file 3: Table S2). During titration, if AEs occurred that might be the result of excessive dopaminergic stimulation, rotigotine could be back-titrated once to the previous dose (and the patient was requested to visit the study site within 1 week for a safety assessment), the patient began the maintenance phase immediately at the back-titrated dose. Dose adjustments were not permitted during maintenance. Clinic visits occurred at screening, baseline, and weeks 1, 2, 3, 4, 5, and 8 of titration/maintenance. Patients who withdrew prematurely were asked to return for a withdrawal visit.

### Outcome measures

The primary variable was safety, as assessed using the Clinical Global Impressions (CGI) item 4 score (side effects) at the end of maintenance: 1 = “None”, 2 = “Not significantly interfering with patient’s functioning”, 3 = “Significantly interfering with the patient’s functioning”, and 4 = “Side effects outweigh therapeutic efficacy”. Other safety evaluations included extent of rotigotine exposure, and treatment-emergent adverse events (AEs, regardless of a causal relationship). In addition, AEs of special interest (those typical of dopaminergic stimulation, use of a transdermal patch, or complications related to PD) were pre-identified and assessed. The AEs of special interest were pre-identified as application site reactions, nausea and vomiting, somnolence, psychosis, sleep attacks/sudden onset of sleep, obsessive-compulsive disorder/impulse-control disorder, postural deformities, freezing gait, and perception disturbances that required atypical antipsychotic treatment. In addition, the modified Minnesota Impulsive Disorder Interview was used to prompt the investigators and patients to monitor the emergence of impulse control disorders. Finally, physical and neurological examinations, changes in laboratory tests, vital signs, 12-lead electrocardiography (ECG), and the Columbia-Suicide Severity Rating Scale (C-SSRS) were also assessed.

Efficacy variables included outcomes assessing motor symptoms, motor fluctuations, non-motor symptoms (sleep disturbance), and quality of life. Change from baseline to end of maintenance was assessed for Unified Parkinson’s Disease Rating Scale (UPDRS) Parts II (activities of daily living [ADL]; mean for the “on” and “off” state), and III (motor examination; in the “on” state); absolute time spent "off" and absolute time spent "on" without troublesome dyskinesia (assessed from patient diaries); Parkinson's Disease Sleep Scale version 2 (PDSS-2); global score of the Pittsburgh Sleep Quality Index (PSQI); Patient Global Impression of Change (PGIC; change from baseline in activity limitations, symptoms, emotions, and overall quality of life; PGIC score 1 = “Very much improved”, 2 = “Much improved”, 3 = “Minimally improved”, 4 = “No change”, 5 = “Minimally worse”, 6 = “Much worse”, 7 = “Very much worse”), and the 8-item short-form Parkinson’s Disease Questionnaire (PDQ-8; assessing PD-related quality of life). Other variables included number of awakenings during the night and number of nocturias, assessed from patient diaries.

### Statistical analyses

The primary variable (CGI item 4 score) was analyzed by the safety set (all enrolled patients who had at least one rotigotine patch applied during the treatment period), using last observation carried forward (LOCF). With the exception of time spent “off”, efficacy variables were analyzed using the full analysis set (FAS; all patients who applied at least one rotigotine patch, and had a baseline and at least one post-baseline UPDRS III assessment), with LOCF. Time spent “off” was analyzed using a subgroup of the FAS comprising patients who recorded time spent “off” at baseline in the patient diary. Efficacy variables were summarized with univariate statistics (mean ± SD), and 95% confidence intervals (CI) were calculated for changes from baseline (i.e., before and after rotigotine add-on).

## Results

### Patient disposition and baseline characteristics

Of 112 patients who provided informed consent and were screened, 22 failed screening, and 90 (80%) applied at least one rotigotine patch and were included in the safety set. Of these 90 patients, 79 (88%) completed the study; the remaining 11 patients (12%) withdrew prematurely due to AEs (n = 5) or for other reasons (n = 6). Baseline demographic data are presented in Table 1.

**Table 1:** **Patient demographics and baseline characteristics, safety set**

	**n = 90**
Age, mean ± SD, years	61.3 ± 9.3
Female, n (%)	43 (48)
Duration of PD, mean ± SD, years	7.4 ± 3.9
Race, n (%)	
Asian	84 (93)
Caucasian	6 (7)
Hoehn and Yahr Stage during “on”, n (%)	
1	1 (1)
2	65 (72)
3	23 (26)
4	1 (1)
Hoehn and Yahr Stage during “off”, n (%)^†^	
2	35 (39)
3	46 (51)
4	7 (8)
Levodopa dosage, mean ± SD, mg/day	547.2 ± 287.7
Oral dopamine receptor agonists dosage, mean ± SD, mg/day	
Pramipexole	0.9 ± 0.5 (n = 51; 57%)
Ropinirole	3.4 ± 2.0 (n = 39; 43%)
Oral dopamine receptor agonist formulation, n (%)	
IR	54 (60)
ER	36 (40)

^†^Data missing from two patients.

*SD*: standard deviation; *PD*: Parkinson’s disease; *IR*: immediate release; *ER*: extended release.

### Safety and tolerability

#### Rotigotine exposure

The mean (±SD) duration of rotigotine exposure was 58.7 ± 14.9 days. A total of 84 patients (93%) entered maintenance. The maintenance dose was 2 mg/24 h in 14 patients, 4 mg/24 h in 19 patients, 6 mg/24 h in 16 patients, and 8 mg/24 h in 35 patients (mean ± SD dose: 5.71 ± 2.28 mg/24 h). Of the 11 patients who discontinued the study, six patients discontinued during the first 2 weeks of titration (at 2 mg/24 h), and the remaining five patients discontinued after between 43 and 56 days (i.e., approx. 6–8 weeks) of rotigotine treatment.

The dose of concomitant oral DA at baseline (as rotigotine converted dose) was 2 mg/24 h in 30 patients, 4 mg/24 h in 29 patients, and 6 mg/24 h in 31 patients (mean ± SD dose: 4.02 ± 1.66 mg/24 h). There was no obvious relationship between the dose of oral DA and dose of rotigotine (Additional file 4: Figure S2).

#### Primary outcome: safety assessed by CGI item 4

Most patients (83/89; 93%) had a score of <3 on CGI item 4 (score of 1: 61/89; 69%, score of 2: 22/89; 25%) at end of treatment, indicating that rotigotine add-on therapy did not interfere with the patient’s functioning. At end of maintenance, a total of six patients (7%) experienced AEs, considered related to study drug by the investigator, that interfered with the patient’s functioning (score ≥3). Three of these six patients were receiving 2 mg/24 h rotigotine at the time of the AE (Table 2).

**Table 2:** **Primary outcome: CGI item 4** ^**†**^ **- Side effects interfering with patient’s functioning (score ≥3); FAS, LOCF**

**Treatment-related AE** ^**‡**^ **(CGI item 4 score)**	**Intensity of AE**	**Rotigotine dose (study phase) at AE onset**	**Action taken with rotigotine**	**Oral DA (actual dose [converted rotigotine dose])**	Total DA dose*	**Levodopa dose mg/day**	**Severity of disease: Hoehn & Yahr Stage during “on”; duration of PD**
Patient 1: Hallucination (score 3)	Moderate	8 mg/24 h (maintenance)	No change	Pramipexole ER (0.75 mg/day [3 mg/24 h])	11 mg/24 h	600	2; 3 years
Patient 2: Dyskinesia (score 4)	Severe	4 mg/24 h (maintenance)	Discontinuation	Ropinirole IR (2 mg/day [2 mg/24 h])	6 mg/24 h	1425	3; 21 years
Patient 3: Nausea (score 3)	Severe	2 mg/24 h (maintenance)	Discontinuation	Pramipexole IR (0.375 mg/day [1.5 mg/24 h])	3.5 mg/24 h	675	3; 10 years
Patient 4: Nausea, dizziness, insomnia, hyperhidrosis (score 4)	Severe	2 mg/24 h (titration)	Discontinuation	Pramipexole IR (1 mg/day [4 mg/24 h])	6 mg/24 h	525	3; 6 years
Patient 5: Rash, dizziness postural (score 3)	Moderate, mild	8 mg/24 h (maintenance)	No change	Pramipexole IR (0.25 mg/day [1 mg])	9 mg/24 h	200	1; 9 years
Patient 6: Worsening of PD (score 4)	Moderate	2 mg/24 h (titration)	Discontinuation	Pramipexole ER (0.75 mg/day [3 mg/24 h])	5 mg/24 h	250	3; 11 years

^†^CGI item 4 score: 1 = none, 2 = not significantly interfering with patient’s functioning, 3 = significantly interfering with the patient’s functioning, 4 = side effects outweigh therapeutic efficacy.

^**‡**^AE considered related to study treatment by the investigator.

*Rotigotine dose at AE onset plus converted rotigotine dose of oral DA.

*AE*: adverse events; *IR*: immediate release; *ER*: extended release; *PD*: Parkinson’s disease; *LOCF*: last observation carried forward; *FAS*: full analysis set.

#### Adverse events

Fifty eight patients (64%) experienced a total of 147 AEs. AEs occurring with an overall incidence of 5% or higher were application site pruritus reported by 12 patients (13%), dizziness (9; 10%), orthostatic hypotension (9; 10%), nausea (7; 8%), dyskinesia (7; 8%), and nasopharyngitis (5; 6%). The incidence of AEs by 1) rotigotine dose, and 2) oral DA dose is presented in Additional file 5: Table S3, and 3) by total DA dose is presented in Additional file 6: Table S4.

Forty four patients (49%) experienced at least one AE during titration, and 25 patients (30%) experienced at least one AE during maintenance. The majority of AEs were mild or moderate in intensity; six patients (7%) experienced at least one severe AE. Six serious AEs were reported in five patients (6%): hallucination, subdural hemorrhage, nasopharyngeal cancer, delirium, confusional state, and urinary retention. Except for the hallucination, all the events were considered unrelated to rotigotine. No deaths were reported.

Five patients were prematurely withdrawn from the study due to AEs (primary reason for withdrawal): dyskinesia (one patient), orthostatic hypotension (one patient), worsening of Parkinson’s disease (one patient), subdural hemorrhage (one patient), dizziness, hyperhidrosis, insomnia, and nausea (one patient). One patient, whose primary reason for withdrawal was due to noncompliance, also discontinued the study due to an AE (nausea). Down-titration of rotigotine was performed in 15 patients due to AEs.

Of the AEs of special interest, 22 (24%) patients experienced application site reactions, eight (9%) nausea and vomiting (seven nausea [preferred term; PT], one vomiting [PT]), four (4%) somnolence (three insomnia [PT], one somnolence [PT]), four (4%) psychosis (two hallucination [PT], one confusional state [PT], one delirium [PT]), and one (1%) patient experienced obsessive-compulsive disorder/impulse-control disorder.

There were no clinically relevant changes in laboratory parameters, vital signs, ECGs, physical and neurological examinations, or the C-SSRS.

### Efficacy

**Figure 1:**
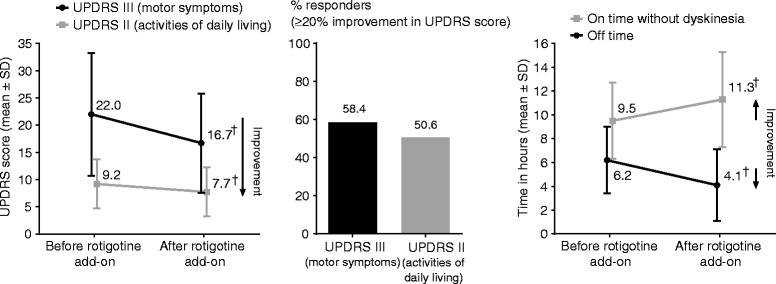
**UPDRS Parts II and III, UPDRS responder analysis, time spent “off”, and time spent “on” with troublesome dyskinesia, FAS, LOCF.** “Before rotigotine add-on”: baseline; “after rotigotine add-on”: end of maintenance. ^†^95% CI does not contain zero (for change from baseline [i.e., before to after rotigotine add-on]). *UPDRS*: Unified Parkinson’s Disease Rating Scale; *FAS*: full analysis set; *LOCF*: last observation carried forward.

Motor function (UPDRS III) and ability to carry out ADL (UPDRS II) was improved following the addition of rotigotine to the existing treatment with low oral DA (Figure 1). Mean (±SD) UPDRS III score (“on” state) at baseline was 22.0 ± 11.3, and change from baseline to end of titration/maintenance was −5.3 ± 8.3 (95% CI: −7.1, −3.6). Mean (±SD) UPDRS II score (average of “on” and “off” states) at baseline was 9.2 ± 4.5, and change from baseline was −1.5 ± 3.8 (95% CI: −2.3, −0.7). The upper limits of 95% CI were below 0, suggesting an improvement in both motor function and patients’ ability to carry out ADL. Improvements were also observed in absolute time spent “off” and absolute time “on” without troublesome dyskinesia (Figure 1); mean (±SD) absolute “off” time change from baseline was −2.1 ± 2.9 h (95% CI: –2.7, –1.5); mean (±SD) absolute time spent “on” without troublesome dyskinesia change from baseline was 1.9 ± 3.1 h (95% CI: 1.2, 2.5). When considering the oral DA formulation, there was no obvious difference in the improvement in time spent “off” between the IR and ER formulations: mean (±SD) absolute “off” time change from baseline was −2.3 ± 3.0 (95% CI: −3.1,–1.4) for patients receiving IR oral DA (baseline 6.4 ± 2.5; n = 46), and −1.9 ± 2.7 (95% CI: −2.8,–0.9) for patients receiving ER oral DA (baseline 6.0 ± 3.2; n = 34).

**Figure 2:**
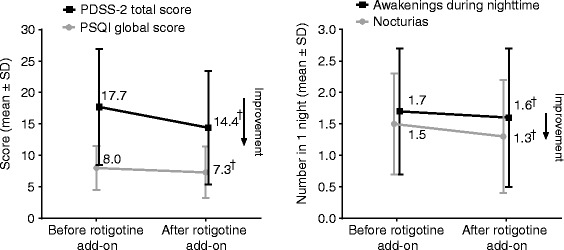
**PDSS-2 total score, PSQI global score, number of awakenings during night time, and number of nocturias, FAS, LOCF.** “Before rotigotine add-on”: baseline; “after rotigotine add-on”: end of maintenance. ^†^95% CI does not contain zero (for change from baseline [i.e., before to after rotigotine add-on]). *PDSS-2*: Parkinson’s Disease Sleep Scale; *PSQI*: Pittsburgh Sleep Quality Index; *FAS*: full analysis set; *LOCF*: last observation carried forward.

**Figure 3:**
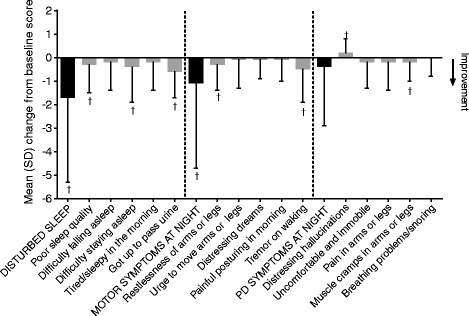
**Change from baseline to end of maintenance in PDSS-2 domain and individual item scores, FAS, LOCF.** ^†^95% CI does not contain zero (for change from baseline [i.e., before to after rotigotine add-on]). *PDSS-2*: Parkinson’s Disease Sleep Scale; *FAS*: full analysis set; *LOCF*: last observation carried forward.

Improvements in items relating to disturbed sleep (a non-motor symptom of PD) were observed, including PDSS-2 total score, PSQI global score, number of awakenings during the night, and number of nocturias (Figure 2); mean (±SD) PDSS-2 total score change from baseline was −3.2 ± 7.5 (95% CI: −4.8, −1.6), and mean PSQI global score change from baseline was −0.7 ± 3.0 (95% CI: −1.4, −0.1). The mean (SD) change from baseline in the number of awakenings during the night was −0.2 ± 0.6 (95% CI: −0.31, −0.04) and for nocturias was −0.2 ± 0.5 (95% CI: −0.3, −0.1). Improvements (i.e., upper limits of 95% CI below 0) were also observed following addition of rotigotine treatment in two of the three domains of the PDSS-2 (“disturbed sleep” and “motor symptoms at night”), and six of 15 individual items. Worsening of the individual PDSS-2 item of “distressing hallucination” was observed (Figure 3).

The majority of patients reported an improvement on the PGIC (71/88; 81%), with 29/88 (33%) reporting “much improved” or “very much improved”, and only 3% (3/88) reporting a deterioration of “much worse” or “very much worse”. Mean (±SD) PDQ-8 total score at baseline was 29.4 ± 17.0, and change from baseline to the end of titration/maintenance was −6.6 ± 14.2 (95% CI: −9.7, −3.6). The upper limit of 95% CI was below 0, suggesting an improvement in items assessing PD-related quality of life.

## Discussion

This open-label study was the first study of rotigotine transdermal system used as an adjunct to treatment with an oral DA. The majority of patients in this study were Hoehn and Yahr stage II-III, and all were inadequately controlled with a treatment regimen of levodopa and low-dose oral pramipexole or ropinirole, presenting with early-morning motor impairment or nocturnal sleep disturbance. The study identified no major safety concerns when rotigotine was added to this treatment regimen. The addition of rotigotine was also associated with numerical improvements in efficacy outcomes, including motor function and sleep disturbances.

The addition of rotigotine was generally well tolerated, with the majority of patients (93%) not experiencing drug-related AEs that interfered with functioning, as assessed by the primary outcome (CGI item 4). In the patients experiencing drug-related AEs that interfered with functioning (CGI item 4 ≥ 3), there was no obvious relationship with the total DA dose (i.e., sum of rotigotine and oral DA dose) or levodopa dose. Of note, the majority of patients experienced an improvement on the PGIC (81%), demonstrating that most patients considered rotigotine add-on therapy as beneficial.

The AE profile was similar to previous studies of rotigotine in patients with advanced PD, with typical dopaminergic side-effects and application site reactions observed, and AEs were comparable with those seen with ropinirole or pramipexole [12-14,18]. They were generally mild or moderate in intensity and led to discontinuation in 6% patients. There was no apparent relationship between the incidence of the most common AEs and the dose of rotigotine, dose of oral DA, or total DA dose. However, as the number of patients receiving the different doses was relatively low, it is not possible to reach conclusions on any potential AE dose-response relationship. Hallucination was reported as an AE in two patients, and the incidence of other dopaminergic AEs including somnolence and impulsive behavior were low (one patient each), and there were no reports of sleep attacks. Therefore, taken together with the CGI item 4 results, the combination of low-dose rotigotine with a low-dose oral DA is likely feasible from a safety perspective.

Numerical improvements in patients’ motor function (UPDRS III) and ability to carry out ADL (UPDRS II) were observed following rotigotine add-on therapy. A reduction in “off” time was observed, with a corresponding prolongation of time spent “on” without troublesome dyskinesia (patient diary). This suggests that the improvement in “on” time was not at the expense of increased dyskinesia. The current study was not designed to investigate the effect of the oral DA formulation (i.e., IR vs ER) on the efficacy outcomes; however, the formulation of the oral DA did not appear to have an obvious effect on the improvement in “off” time. Improvements in the non-motor symptom of sleep disturbance (PDSS-2, PSQI, and patient diaries), and PD-related quality of life (PDQ-8) were also observed.

In some patients with PD, symptoms may not be adequately controlled with existing oral DA treatment, and the dose cannot be sufficiently increased due to adverse drug reactions. In addition, in some cases, night-time symptoms may be observed although the current dose of DA improves day-time symptoms. In the present study, the patients were taking seemingly low doses of pramipexole/ropinirole, yet they were receiving stable doses which were maximal for the individual (i.e., “best” pharmacological therapy). The patients were inadequately controlled on these doses, and the addition of rotigotine aimed to further control their symptoms. We observed that concurrent activation of D2 and D3 receptors following dual DA therapy was feasible, at least in the dose ranges used. However, the mechanism/s by which rotigotine added to pramipexole or ropinirole induced potential benefits on efficacy outcomes (e.g., motor control, fluctuations, sleep disturbance) can only be speculated. They may result from the increase in DA total dose (i.e., leading to more robust activation of D2/D3 receptors), the different DA receptor profiles (i.e., stimulation of D1 receptor, which is unique to rotigotine, combined with D2/D3 activation) and/or the different pharmacokinetics.

There are some potential limitations to consider. First, as there was no comparator arm or control group, and no statistical significance testing was performed, this limits the conclusions that can be drawn. Second, the ability to generalize the results of this study is restricted by the entry criteria of the study, and so limited to patients with advanced PD inadequately controlled on levodopa and a low-dose oral DA. Third, longer term safety cannot be concluded from this 8-week study. Finally, to prevent potential overstimulation of D2/D3 receptor after concomitant use of rotigotine-pramipexole and rotigotine-ropinirole, less than half of the respective approved maximum doses were used in this study. Therefore, we cannot conclude whether higher doses may provide further benefits or be associated with safety concerns.

## Conclusions

In summary, this study demonstrates that addition of rotigotine transdermal system to a low-dose oral DA was feasible and may be associated with clinical benefit in patients with advanced PD inadequately controlled on levodopa and a low-dose oral DA. Dual therapy with rotigotine transdermal system and a low-dose oral DA in PD may represent a promising approach to treatment. Equivalent dose should be taken into consideration when the DAs are used concomitantly, and the maximum dose of DAs should not exceed the upper limit of the approved dose of any of the DAs. Double-blind controlled studies are required to determine the significance and clinical relevance of these findings.

## Additional files

Additional file 1: Table S1. Institutional Review Board review results at each trial site in the PD0015 study. Additional file 2: Figure S1. Study design. Additional file 3: Table S2. General recommendations for equivalent dosing regimens. Doses in bold show permitted maximum dose of each dopamine receptor agonist in the PD0015 study. ^†^Pramipexole doses are expressed in terms of pramipexole dihydrochloride monohydrate (pramipexole salt); 1.0 mg pramipexole salt corresponds to 0.7 mg of pramipexole monohydrate (pramipexole base). Additional file 4: Figure S2. Oral DA and rotigotine dose distribution, safety set. ^†^Converted rotigotine dose. Dose of rotigotine presented by dose at end of titration. *DA*: dopamine receptor agonist. Additional file 5: Table S3. Adverse events (AEs) (titration/maintenance phase) occurring in at least 5% of patients^†^ reported by rotigotine and oral DA dose. ^†^AEs occurring in at least 5% of all patients during the entire study. ^‡^Converted rotigotine dose. *DA*: dopamine receptor agonist. Additional file 6: Table S4. Adverse events (AEs) (titration/maintenance phase) occurring in ≥5% of patients^†^ reported by total DA dose. ^†^AEs occurring in at least 5% of all patients during the entire study. ^‡^Converted rotigotine dose. *DA*: dopamine receptor agonist.
