# Supplementary material for: Rotigotine transdermal system as add-on to oral dopamine agonist in advanced Parkinson’s disease: an open-label study
Source: BMC Neurol. 2015 Feb 28;15:17. doi: 10.1186/s12883-015-0267-7 (PMC4364324; doi:10.1186/s12883-015-0267-7)
Supplement: Additional file 1: Table S1. — Institutional Review Board review results at each trial site in the PD0015 study. [file 12883_2015_267_MOESM1_ESM.docx]

**Additional file 1: Table S1. Institutional Review Board review results at each trial site in the PD0015 study.**

| **Site** | **Date of IRB meeting**  **(or expedited review)** | **Date of approval**  **(Conditions)** |
| --- | --- | --- |
| **Korea** | | |
| 101. Severance Hospital | 20/Jul/2012,  13/Sep/2012 | 20/Jul/2012 (Conditional approval) 13/Sep/2012 (Approval) |
| 102. Gangnam Severence Hospital | 11/Jul/2012 | 11/Jul/2012 (Approval) |
| 103. Asan Medical Center | 06/Aug/2012 | 06/Aug/2012 (Approval) |
| 104. Inje University Busan Paik Hospital | 19/Jul/2012 | 19/Jul/2012 (Approval) |
| 105. Samsung Medical Center | 25/Aug/2012  31/Oct/2012 | 25/Aug/2012 (Conditional approval) 31/Oct/2012 (Approval) |
| 106. Kyunghee University Hospital | 12/Jul/2012  24/Sep/2012  13/Nov/2012 | 12/Jul/2012 (Conditional approval) 24/Sep/2012 (Approval with recommendation) 13/Nov/2012 (Approval) |
| 107. Hanyang University Seoul Hospital | 31/Jul/2012  25/Sept/2012 | 31/Jul/2012 (Conditional approval) 25/Sep/2012, (Approval) |
| 108. Korea University Anam Hospital | 06/Aug/2012 | 06/Aug/2012 (Approval) |
| 109. Kyungpook National University Medical Center | 14/Aug/2012 | 14/Aug/2012 (Approval) |
| 110. Seoul National University Hospital | 02/Aug/2012  04/Oct/2012 | 02/Aug/2012 (Conditional approval) 04/Oct/2012 (Approval) |
| 111. Seoul University Bundang Hospital | 23/Jul/2012  28/Aug/2012 | 23/Jul/2012 (Conditional approval) 28/Aug/2012 (Approval) |
| 112. Dong-A University Medical Center | 25/Jul/2012  30/Aug/2012 | 25/Jul/2012 (Conditional approval) 30/Aug/2012 (Approval) |
| **Malaysia** | | |
| 201. Hospital Seberang Jaya  202. Hospital Sultanah Nur Zahirah  204. Hospital Umum Sarawak | 24/Jul/2012 | 18/Sep/2012 |
| **Taiwan** | | |
| 302. China Medical University Hospital | 12/Sep/2012 | 20/Oct/2012 |
| 303. Chi-Mei Medical Center | 13/Sep/2012 | 13/Sep/2012 |
| 306. National Taiwan University Hospital | 14/Sep/2012 | 17/Oct/2012 |

| **Australia** | | |
| --- | --- | --- |
| 401. Southern Neurology, St George Private Hospital (Sydney)  402. Strategic Health Evaluators (SHE)  403. Flemington Neurology | 29/Aug/2012 | 22/Oct/2012 (401)  06/Nov/2012 (402)  30/Oct/2012 (403) |
| 404. St Vincent’s Hospital (NSW)  407. Concord Hospital (Sydney) | 22/Nov/2012 | 22/Nov/2012 |
| **Singapore** | | |
| 501. National Neuroscience Institute  502. Singapore General Hospital | 28/Sep/2012 | 4/Nov/2012 |
