# Supplementary material for: Rotigotine transdermal system as add-on to oral dopamine agonist in advanced Parkinson’s disease: an open-label study
Source: BMC Neurol. 2015 Feb 28;15:17. doi: 10.1186/s12883-015-0267-7 (PMC4364324; doi:10.1186/s12883-015-0267-7)
Supplement: Additional file 3: Table S2. — General recommendations for equivalent dosing regimens. Doses in bold show permitted maximum dose of each dopamine receptor agonist in the PD0015 study. †Pramipexole doses are expressed in terms of pramipexole dihydrochloride monohydrate (pramipexole salt); 1.0 mg pramipexole salt corresponds to 0.7 mg of pramipexole monohydrate (pramipexole base). [file 12883_2015_267_MOESM3_ESM.docx]

**Supplemental Table 2** **General recommendations for equivalent dosing regimens**

| Drug | Equivalent dose (mg/24 h) | | | | | | | |
| --- | --- | --- | --- | --- | --- | --- | --- | --- |
| Pramipexole [4]^†^ | 0.5 | 1 | 1.5 | 2 | 2.5 | 3 | 3.5 | 4 |
| Ropinirole [4, 17] | 2 | 4 | 6 | 8 | 10 | 12 | 14 | 16 |
| Rotigotine [12, 17] | 2 | 4 | 6 | 8 | 10 | 12 | 14 | 16 |

Doses in bold show permitted maximum dose of each dopamine receptor agonist in the PD0015 study.

^†^Pramipexole doses are expressed in terms of pramipexole dihydrochloride monohydrate (pramipexole salt); 1.0 mg pramipexole salt corresponds to 0.7 mg of pramipexole monohydrate (pramipexole base)
