# Supplementary figures and images for: Rotigotine transdermal system as add-on to oral dopamine agonist in advanced Parkinson’s disease: an open-label study
Source: BMC Neurol. 2015 Feb 28;15:17. doi: 10.1186/s12883-015-0267-7 (PMC4364324; doi:10.1186/s12883-015-0267-7)

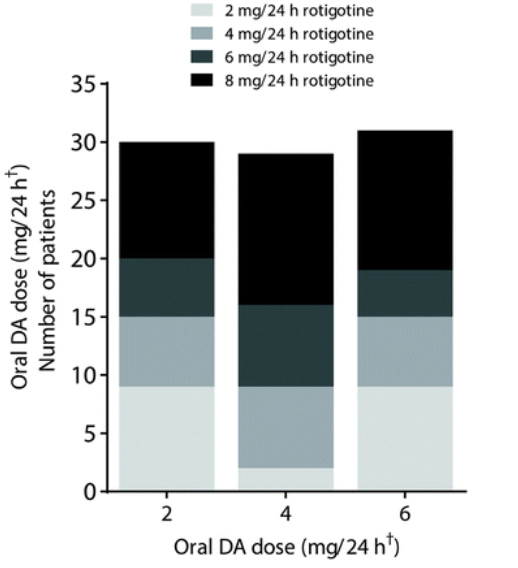

Supplement: Additional file 4: Figure S2. — Oral DA and rotigotine dose distribution, safety set. †Converted rotigotine dose. Dose of rotigotine presented by dose at end of titration. DA: dopamine receptor agonist. [file 12883_2015_267_MOESM4_ESM.docx]
