# Supplementary material for: Rotigotine transdermal system as add-on to oral dopamine agonist in advanced Parkinson’s disease: an open-label study
Source: BMC Neurol. 2015 Feb 28;15:17. doi: 10.1186/s12883-015-0267-7 (PMC4364324; doi:10.1186/s12883-015-0267-7)
Supplement: Additional file 5: Table S3. — Adverse events (AEs) (titration/maintenance phase) occurring in at least 5% of patients† reported by rotigotine and oral DA dose. †AEs occurring in at least 5% of all patients during the entire study. ‡Converted rotigotine dose. DA: dopamine receptor agonist. [file 12883_2015_267_MOESM5_ESM.docx]

**Additional file 5: Table S3. Adverse events (AEs) (titration/maintenance phase) occurring in at least 5% of patients^†^ reported by rotigotine and oral DA dose**

|  | Rotigotine dose at end of titration (mg/24 h) | | | | Oral DA dose (mg/24 h)^‡^ | | |
| --- | --- | --- | --- | --- | --- | --- | --- |
| n (%) | 2 n = 20 | 4 n = 19 | 6 n = 16 | 8n = 35 | 2 n = 30 | 4 n = 29 | 6 n = 31 |
| Any AE | 14 (70) | 12 (63) | 11 (69) | 18 (51) | 21 (70) | 18 (62) | 16 (52) |
| Application site pruritus | 1 (5) | 3 (16) | 4 (25) | 4 (11) | 3 (10) | 5 (17) | 4 (13) |
| Dizziness | 2 (10) | 2 (11) | 2 (13) | 3 (9) | 3 (10) | 5 (17) | 1 (3) |
| Orthostatic hypotension | 1 (5) | 2 (11) | 3 (19) | 2 (6) | 1 (3) | 2 (7) | 5 (16) |
| Nausea | 4 (20) | 1 (5) | 1 (6) | 1(3) | 4 (13) | 2 (7) | 1 (3) |
| Dyskinesia | 3 (15) | 3 (16) | 1 (6) | 0 | 4 (13) | 0 | 3 (10) |
| Nasopharyngitis | 2 (10) | 0 | 1 (6) | 1 (3) | 1 (3) | 1 (3) | 2 (6) |

^†^AEs occurring in at least 5% of all patients during the entire study.

^‡^Converted rotigotine dose.

*DA*: dopamine receptor agonist.
