# Supplementary material for: Rotigotine transdermal system as add-on to oral dopamine agonist in advanced Parkinson’s disease: an open-label study
Source: BMC Neurol. 2015 Feb 28;15:17. doi: 10.1186/s12883-015-0267-7 (PMC4364324; doi:10.1186/s12883-015-0267-7)
Supplement: Additional file 6: Table S4. — Adverse events (AEs) (titration/maintenance phase) occurring in ≥5% of patients† reported by total DA dose. †AEs occurring in at least 5% of all patients during the entire study. ‡Converted rotigotine dose. DA: dopamine receptor agonist. [file 12883_2015_267_MOESM6_ESM.docx]

**Additional file 6: Table S4. Adverse events (AEs) (titration/maintenance phase) occurring in ≥5% of patients^†^ reported by total DA dose**

| Adverse event | Total DA dose:Sum of rotigotine dose at end of titration and oral DA dose^‡^ (mg/24 h) | | | | | |
| --- | --- | --- | --- | --- | --- | --- |
|  | 4 (n = 9) | 6 (n = 8) | 8(n = 21) | 10 (n = 23) | 12 (n = 17) | 14 (n = 12) |
| Any AE | 6 (67) | 6 (75) | 14 (67) | 16 (70) | 9 (53) | 4 (33) |
| Application site pruritus | 1 (11) | 0 | 2 (10) | 6 (26) | 1 (6) | 2 (17) |
| Dizziness | 1 (11) | 1 (13) | 1 (5) | 5 (22) | 1 (6) | 0 |
| Orthostatic hypotension | 0 | 0 | 2 (10) | 4 (17) | 0 | 2 (17) |
| Nausea | 3 (33) | 1 (13) | 1 (5) | 1 (4) | 1 (6) | 0 |
| Dyskinesia | 1 (11) | 2 (25) | 3 (14) | 1 (4) | 0 | 0 |
| Nasopharyngitis | 1 (11) | 0 | 1 (5) | 0 | 2 (12) | 0 |

^†^AEs occurring in at least 5% of all patients during the entire study

^‡^Converted rotigotine dose.

*DA:* dopamine receptor agonist.
