# Supplementary material for: Rotigotine transdermal system as add-on to oral dopamine agonist in advanced Parkinson’s disease: an open-label study
Source: BMC Neurol. 2015 Feb 28;15:17. doi: 10.1186/s12883-015-0267-7 (PMC4364324; doi:10.1186/s12883-015-0267-7)
Supplement: Supplementary file 6 [file 12883_2015_267_MOESM2_ESM.docx]

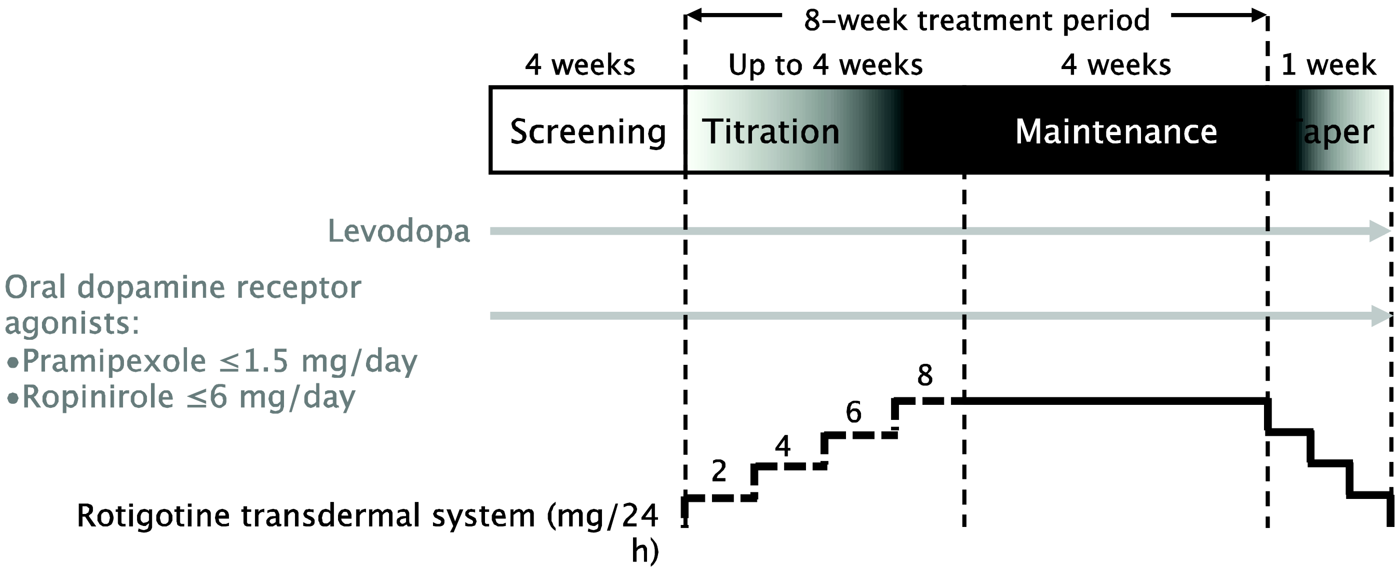

8-week treatment period
4 weeks
Up to 4 weeks
4 weeks
1 week
Screening
Titration
Maintenance
Taper
Levodopa
Oral dopamine receptor agonists:
•Pramipexole ≤1.5 mg/day
•Ropinirole ≤6 mg/day
2
4
6
8
Rotigotine transdermal system (mg/24 h)
